# Telehealth autism evaluation under suboptimal network conditions: Technical parameters and clinical implications

**DOI:** 10.1371/journal.pone.0357146

**Published:** 2026-09-09

**Authors:** Senad Kolar, Tomas Horvath, Lucie Stroupkova, Martina Vyhnalova, Anna Marie Bittnerova, Nela Novakova, Ivona Packanova, Lenka Knedlikova, Rastislav Repko, Petr Munster, Pavlina Danhofer

**Affiliations:** 1 Department of Pediatric Neurology, Faculty of Medicine, Masaryk University and University Hospital Brno, Brno, Czech Republic; 2 Department of Telecommunications, Brno University of Technology, Brno, Czech Republic; 3 Faculty of Medicine, Masaryk University, Brno, Czech Republic; Father Muller Charitable Institutions, INDIA

## Abstract

Telehealth expands access to autism diagnostics, but scoring reliability depends on connection stability. Using an optical-fiber testbed, we imposed controlled packet loss and Wi-Fi attenuation to derive four freeze patterns (1-second freezes every 16.7, 11.1, 8.3, or 5.0 seconds), then applied these to 10 children’s BOSA videos (40 clips) scored by four blinded psychologists. Analyses included Fisher’s exact tests with Benjamini–Hochberg correction, Spearman correlation, and per-item logistic regression with Bonferroni correction. Performance degraded in a dose-dependent manner rather than collapsing: below a Received Signal Strength Indicator (RSSI) of −70 dBm or above ~0.04% packet loss, freeze frequency increased from ~1/16.7 s to ~1/5.0 s. Time-sensitive items–Spontaneous Joint Attention (B10), Integration of Gaze and Behavior (B4), Immediate Echolalia (A4), and Stereotyped/Idiosyncratic Language (A5)–were 4–9 × more likely to be coded unscorable, and poorer stream quality correlated with more unscored items (ρ ≈ −0.58). Importantly, overall BOSA scoring remained stable, with no individual item reaching significance after correction and substantial inter-rater consistency under mild degradation (ICC = 0.93, Cohen’s κ = 0.76), indicating robustness to moderate degradation. In this pilot study, these results support BOSA as a first-line option when packet loss remains below ~0.04% and RSSI stays above −70 dBm, pending validation in larger, more diverse cohorts. Where possible, using wired connections and awareness of item-level sensitivities can further preserve diagnostic confidence.

## 1. Introduction

Telehealth has rapidly evolved from a pandemic-driven solution into a routine method for behavioral assessment, including autism diagnostics. Video-mediated assessments such as the Brief Observation of Symptoms of Autism (BOSA) and tele-adapted ADOS (Autism Diagnostic Observation Schedule) protocols now allow clinicians to reach families who would otherwise face long travel times or specialist wait-lists [1,2]. Yet the diagnostic integrity of these remote tools still depends on something decidedly non-clinical: the stability of the end-user’s internet link. Even brief packet loss or Wi-Fi attenuation can freeze a child’s face at the very moment joint attention or echolalia needs to be judged [3,4]. Recent surveys place “poor connection quality” among the top three reasons clinicians abandon or reschedule tele-autism visits [5], but there is sparse empirical guidance on how much loss is too much, which behavioral items collapse first, or what simple metrics families can check before a call begins.

Existing studies tend to describe bandwidth qualitatively (“adequate”, “stable”) or report average speeds without linking them to item-level scoring reliability [6,7]. Consequently, programs lack useful cut-offs to decide when a home visit can proceed, when to deploy technical support, or when to re-route a family to a bandwidth-assured hub. Moreover, codec improvements and adaptive bit-rate algorithms used by common platforms (e.g., Zoom, Webex) mean that raw download speed is no longer the only–or even the best–predictor of diagnostic risk; packet-loss, latency spikes and Wi-Fi signal power may matter more.

This study addresses that gap by combining controlled laboratory measurements with a clinical validation arm. We began by empirically mapping Zoom’s operating limits under degraded-network conditions, then we emulated real-world Wi-Fi fade and packet-drop patterns to define four graded degradation scenarios. Using a custom Python script, we imposed identical freeze intervals on BOSA recordings from a pediatric neurology cohort and tracked how often specific items become unscorable. By mapping freeze frequency and packet-loss to item-level failure, we aim to:

- Establish quantitative network thresholds–in dBm signal strength and packet-loss rate–above which BOSA scoring remains reliable.
- Identify which types of behavior (e.g., spontaneous joint attention, immediate echolalia) are most vulnerable to video freeze.

By defining the vague term “adequate connection” as measurable numbers and workflow triggers, we hope to equip clinicians, families and payers with evidence-based criteria that keep remote autism evaluation both accessible and diagnostically valid.

## 2. Methods

**Fig 1:**
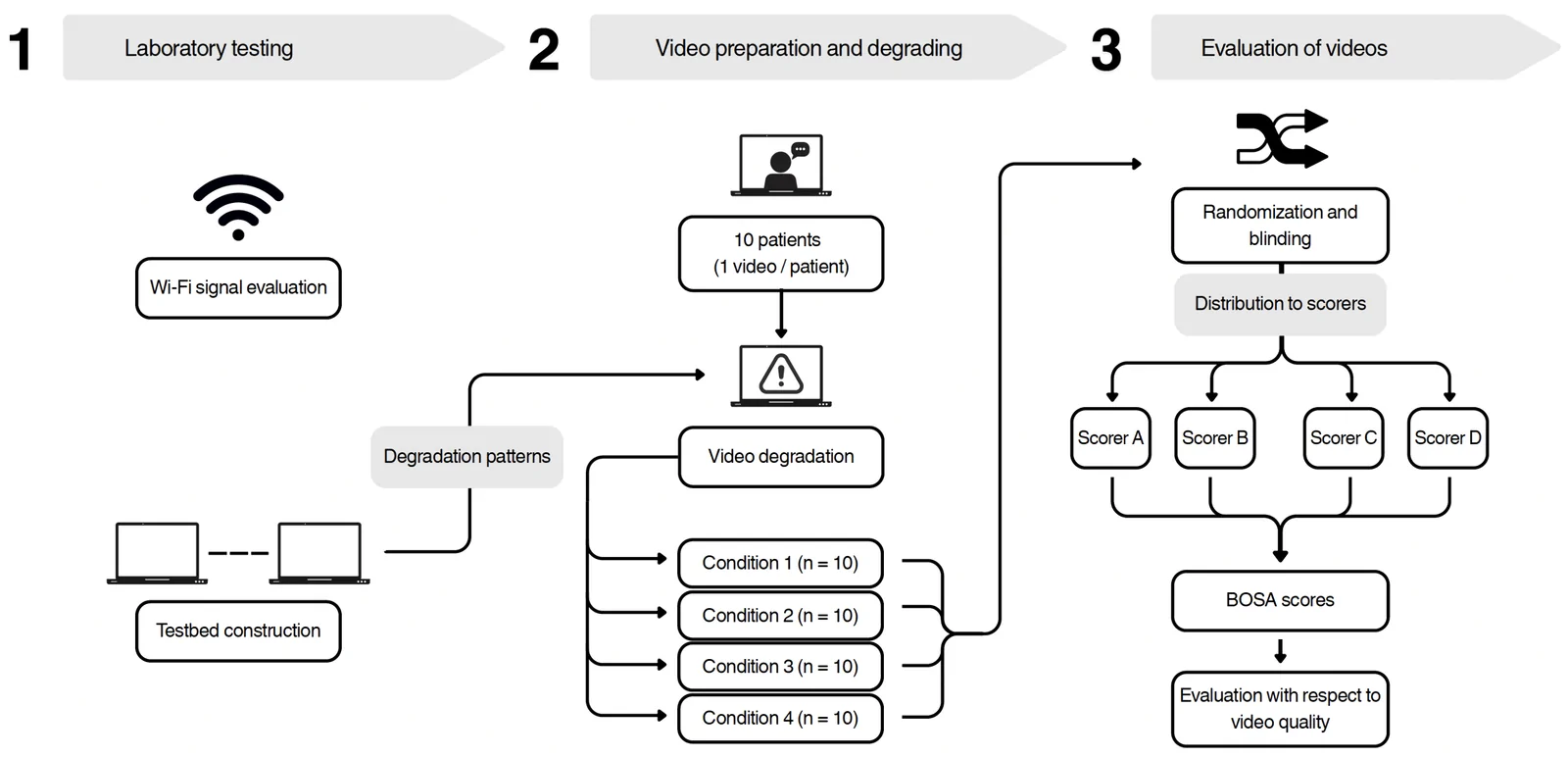
Overview of study workflow. The process had three phases: (1) Laboratory testing, including Wi-Fi signal evaluation and construction of a controlled testbed to define degradation patterns; (2) Video preparation and degrading, where 10 patient BOSA videos were systematically degraded into four freeze-rate conditions; and (3) Evaluation of videos, in which clips were randomized, blinded, distributed to four scorers, and assessed for BOSA scoring reliability relative to video quality.

The study follows three phases (Fig 1). First, we benchmarked Zoom on matched laptops connected through an optical-fiber testbed while injecting precisely titrated packet delay, drop and duplication. Wi-Fi walk-tests in a standard apartment establish the signal-strength breakpoint where freeze frequency exceeds clinical tolerance. The output is a set of four degradation “steps” (freeze every 5 s, 8.3 s, 11.1 s or 16.7 s) implemented in a Python script. Second, full-HD BOSA recordings from 10 in-patient children (age 2–8 years; one per child) were rendered into each degradation condition (40 clips total). Third, clips were randomized, blinded, and distributed to four psychologists for scoring per BOSA rules (with “8” for unscorable). Analyses included item-level observed vs expected counts (Fisher’s exact, Benjamini–Hochberg), association between degradation and unscorable totals (Spearman ρ), and item scorability (logistic regression).

### 2.1. Parameter acquisition for video degradation

#### 2.1.1. Laboratory testing of network parameters.

a) Hardware and Connectivity Requirements Analysis

We conducted a comprehensive analysis of Zoom videoconferencing requirements. Hardware specifications were documented, including minimum (2 GHz dual-core CPU, 4 GB RAM) and recommended configurations (quad-core 2.5 GHz CPU, 16 GB RAM). For one-on-one telehealth interactions, bandwidth requirements ranged from 600 kbps for basic video quality to 3.8 Mbps for 1080p resolution, with all configurations requiring symmetrical upload and download speeds.

b) Wi-Fi signal threshold determination

To simulate real-world clinical scenarios, we established an experimental setup where the clinician position remained fixed while the “patient” position varied throughout a standard apartment environment. This approach prevented testing bias toward ideal connectivity scenarios. At each location, we conducted a systematic assessment of:

- Video transmission quality (subjective evaluation of stuttering, pixelation, and freezing)
- Wi-Fi signal power measurements (dBm)
- Correlation between signal degradation and communication breakdowns
- Effectiveness of Zoom’s dynamic resolution adjustment features

The experiments focused on fixed broadband connections due to customer-side constraints, such as Wi-Fi router limitations, signal interference, and coverage issues. While 5G mobile networks must be considered a viable connectivity alternative, their integration faces challenges in establishing reproducible testing conditions [8,9]. This difficulty arises from end-users lacking real-time data on local gNodeB utilization levels, leading to inconsistent network load scenarios across experiments.

c) Isolated Testbed Network Parameter Testing

We constructed a controlled isolated testbed environment to systematically evaluate network disruption effects (S1 Fig):

- Two laptops meeting Zoom’s minimum hardware specifications
- Media converters linked via single-mode optical fiber link
- VLC players for video streaming of pre-recorded clinical footage
- “Clumsy 0.3” application for precise network disruption simulation

The isolated approach was specifically designed to maintain patient confidentiality while allowing systematic parameter manipulation. Three network disruption scenarios were rigorously tested:

1. Lagging: Systematic packet delays at specified intervals
2. Dropping: Controlled random packet loss at precise percentages
3. Duplicating: Packet cloning to analyze redundancy effects

For each scenario, we documented transmission quality, artifact patterns, and clinical usability thresholds. Packet dropping demonstrated the most significant clinical impact and was selected for detailed parametric analysis, as it produced the discrete frame-freeze patterns most disruptive to behavioral observation (S1 Table). The four freeze intervals subsequently used in the experimental phase were not chosen arbitrarily, but derived directly from packet-loss rates measured on this testbed: each interval corresponds to a specific packet-loss percentage at which Zoom’s adaptive codec produced a characteristic freeze frequency under repeatable conditions, combined with subjective evaluation of clinical usability at each level.

d) Custom Video Degradation Implementation

Based on laboratory findings, we developed and validated a Python-based video processing tool using the *MoviePy* library. The script systematically partitions input videos into precisely timed segments, applies freeze-frame effects at configurable intervals with a fixed freeze duration, processes multiple videos with consistent degradation patterns, and produces output files with embedded parameter metadata.

The final degradation script used fixed freeze intervals and a constant freeze duration rather than randomly distributed interruptions. While real networks produce temporally irregular packet loss that varies with connection technology, instantaneous load, and signal quality, a pseudorandom implementation would have introduced additional dependencies on scene content, frame structure, and system load, severely limiting cross-clip comparability. Fixed intervals were therefore chosen to ensure reproducibility and direct comparison across videos and degradation levels. The four specific freeze rates applied in the experimental phase were derived empirically from the testbed measurements described above, combined with subjective evaluation of video usability at each packet-loss level. Because the script was applied to complete audiovisual files, audio and video degradation occurred simultaneously in the simulated clips, whereas Zoom processes audio (Opus/SILK codecs) and video (H.264 SVC) independently and prioritizes audio under packet loss in live sessions.

### 2.2. Experimental part

Based on findings from the laboratory testing, we designed an experimental validation study to systematically evaluate how video quality degradation impacts the reliability of autism assessments. This experimental component utilized video freezing patterns to simulate real-world network limitations observed in telehealth settings. We created a controlled testing environment where clinical videos were subjected to precisely defined quality degradation levels, enabling us to analyze specific relationships between video quality and assessment outcomes.

a) Participants and procedure – This single-center study recruited participants from hospitalized patients at the Department of Pediatric Neurology between 1 April 2024 and 31 March 2025. These patients were hospitalized due to autism or speech delay assessment. Every patient was tested with ADOS-2 and subsequently with BOSA. During BOSA, the parent of the participant exposed the child to stimulus situations. The psychologist responsible for the BOSA procedure observed the administration through a one-way mirror from another room and gave the instructions using sound equipment in the rooms. BOSA procedure was filmed using a Panasonic GX9 DSLR camera in full-HD resolution (1920x1080 px) using the internal microphone.

The study was approved by the Institutional Review Board of University Hospital Brno (protocol no. 36/2024) and by the Ethics Committee of the Faculty of Medicine, Masaryk University (protocol no. 04/2024). Written informed consent for participation and video recording was obtained from the parents or legal guardians of all participants, after the study procedures were explained in detail. All data were handled in a pseudonymized manner. Researchers from the Department of Telecommunications signed a nondisclosure agreement and worked exclusively with anonymized video material. No identifiable data were accessible during or after analysis.

Information about patients, including age, sex, ADOS-2 scores and undegraded BOSA scores, is shown in Table 1.

**Table 1:** Participant characteristics and ADOS-2 baseline. Sex, age (years), clinical diagnosis (ASD vs speech delay), and ADOS-2 scores are shown: Algorithm total, Comparison Score, and domain totals for SA (Social Affect) and RRB (Restricted and Repetitive Behaviors).

ID	Sex	Age (years)	Diagnosis	Algorithm Score	Comparison Score	SA Social Affect Domain	RRB – Restricted Repetitive Behavior Domain
1	M	6	Speech delay	20	7	17	3
2	M	7	ASD	26	10	20	6
3	M	3	ASD	26	10	18	8
4	M	8	ASD	13	6	7	6
5	M	3	Speech delay	4	2	4	0
6	M	2	ASD	13	4	11	2
7	F	6	ASD	24	8	20	4
8	F	2	ASD	25	10	20	5
9	M	7	ASD	10	5	7	3
10	F	6	ASD	26	10	20	6

b) Video degradation – using a custom Python script, we introduced frame freezes to the clinical footage to elicit varying network conditions. Based on values from laboratory testing, we decided to use the following freeze rates (always for one second):
  - Condition 1: Video freezes every 5 seconds
  - Condition 2: Video freezes every 8.33 seconds
  - Condition 3: Video freezes every 11.11 seconds
  - Condition 4: Video freezes every 16.67 seconds

Each clinical video was degraded with all the conditions, so the final count for conditions was 10 for each of them – Condition 1–4.

c) BOSA assessment – each degraded video was then distributed to one of 4 examiners (clinical psychologists) for evaluation. Psychologists were blinded about the information whether the respective subject had ASD and to the fact that the video footage had been degraded.

Psychologists then proceeded with the assessment according to BOSA instructions. If an item could not be scored, it was marked with code 8. We assume that a code 8 could have been assigned for three reasons: (1) video quality issues, (2) absence of the relevant situation in which the behavior could be observed (e.g., the participant was turned away from the camera, so eye contact with the parent could not be evaluated), or (3) requirements specified in the ADOS-2/BOSA scoring system. The total BOSA score was then calculated by summing the core items critical for identifying ASD.

d) Data analysis

To evaluate the impact of video stream quality on scoring accuracy, we first needed to determine which BOSA items were disproportionately affected by video quality. For each item, we calculated:

- Observed frequency: The actual count of unscored entries (marked as ‘8’) for each item
- Expected frequency: What would be expected if all items were equally affected by video quality

To calculate expected frequencies, we used a baseline derived from the overall unscored rate across all other items. To do that, we:

- Calculated the average unscored rate across all other items for each participant group.
- Multiplied this rate by the number of participants, yielding the expected number of unscored entries by chance.

We then compared this expected count to the observed count using Fisher’s exact test while adjusting for multiple comparisons using the Benjamini-Hochberg procedure to control the false discovery rate. This approach allowed us to identify items that were significantly more likely to be unscored than would be expected based on the general “background rate” of unscored entries, suggesting these items were particularly vulnerable to video quality deterioration.

For these vulnerable items, we compared unscored frequencies between poor and better stream quality groups using Fisher’s exact test. We also employed Spearman’s rank correlation to assess the relationship between stream quality conditions and total number of unscored items across all participants.

Lastly, we used binary logistic regression to evaluate whether stream quality (Code 1–4, ordinal) predicted the likelihood of items being unscored (binary outcome). Models were fitted separately for each item with stream quality as the sole predictor. Of the 25 BOSA items analyzed, four (B14, B15, D3, E1) were excluded due to perfect separation, where stream quality perfectly predicted scoring status, leaving 21 items in the final analysis. As each model included a single predictor, model convergence was confirmed for all 21 retained items. Given the small sample size and the sparseness of unscored events per item, formal goodness-of-fit testing was not pursued. Odds ratios are reported with 95% confidence intervals and unadjusted and Bonferroni-adjusted p-values for all 21 items (S2 Table), with statistical significance set at adjusted p < 0.05; intervals were wide, reflecting the limited number of unscored events per item, and results are therefore interpreted as exploratory.

## 3. Results

### 3.1. Parameter acquisition for video degradation

#### 3.1.1. Laboratory testing of network parameters.

a) Wi-Fi Signal Threshold Identification

Wi-Fi signal testing revealed a critical threshold at approximately –70 dBm, below which significant video quality degradation occurred. Signal strength ranged from optimal (–42 dBm near router) to severely degraded (–75 dBm at maximum distance). When signals dropped below the –70 dBm threshold, video freezing became the predominant issue, triggering Zoom’s “unstable connection” notifications and interrupting clinical assessment despite the application’s dynamic resolution adjustment capabilities.

b) Packet Loss Impact on Video Quality

The packet dropping tests revealed precise relationships between packet loss rates and video freezing patterns, enabling us to establish four experimental conditions:

- Condition 1 (5-second freeze interval): Corresponds to approximately 0.04% packet loss rate characterized by minor artifacts affecting up to one-third of the image, with unpredictable distortion locations. This level represents the threshold at which facial expressions become difficult to interpret, and clinical assessment reliability is potentially compromised.
- Condition 2 (8.33-second freeze interval): Approximately maps to a 0.035% packet loss rate, where minor artifacts appear irregularly across up to three-quarters of the image. At this level, rapid movements create noticeable blur but faces generally remain recognizable during static periods.
- Condition 3 (11.11-second freeze interval): Closely corresponds to the 0.03% packet loss rate, creating periodic artifacts distributed across the video frame with improved stability between freezes. This condition allows for generally reliable facial recognition with occasional assessment interruptions.
- Condition 4 (16.67-second freeze interval): Represents approximately 0.025% packet loss, where artifacts affect up to one-quarter of the image irregularly. This level permits relatively stable clinical assessment with minimal disruption to behavioral observation.

The experimental validation demonstrated that complete clinical unusability occurred at packet loss rates exceeding 0.15%, where consistent artifacts and image freezing rendered up to two-thirds of video content unrecognizable.

c) Video Degradation Script Implementation

The custom Python script successfully converted our laboratory findings into precisely controlled simulation conditions, allowing for consistent application of freeze-frame effects at the four specified intervals with a constant 1-second freeze duration, batch processing of clinical assessment videos, preservation of video quality parameters between freeze events, and integration of metadata for experimental condition tracking. This methodology established a robust foundation for subsequent clinical validation of telehealth assessment reliability under varying network conditions.

### 3.2. Experimental part

The last part of the analysis was to use prepared degraded videos to examine how video quality degradation during telehealth assessments affects BOSA item scoring. First, we assessed which BOSA items are most susceptible to stream quality issues. Then we evaluated differences between poor and better stream quality groups on vulnerable items. We then looked to quantify the overall relationship between stream quality and unscored items. Lastly, we determined the influence of stream quality on total BOSA score.

#### 3.2.1. Identifying BOSA items vulnerable to video quality issues.

We first identified which specific BOSA items were disproportionately affected by stream quality issues by comparing observed versus expected frequencies of unscored items. This approach allowed us to isolate vulnerable assessment components regardless of specific stream quality groups.

Four BOSA items showed significantly higher unscored rates than statistically expected – B10, Spontaneous Initiation of Joint Attention; B4, Integration of Gaze and Other Behaviors; A4, Immediate Echolalia; and A5, Stereotyped/Idiosyncratic Use of Words/Phrases. Odds ratios, confidence intervals and p values can be found in Table 2.

**Table 2:** Comparison of observed vs. expected unscored frequencies for BOSA items. The items listed above were significantly more likely to be unscored than expected, independent of specific stream quality groups, reflecting their vulnerability to technical challenges.

Item	Observed Unscored	Odds Ratio	95% CI	Adjusted p-value	Significant
**B10. Spontaneous Initiation of Joint Attention**	14	7.89	3.80, 16.36	<0.0001	Yes
**B4. Integration of Gaze and Other Behaviors During Social Overtures**	11	5.15	2.42, 10.94	0.0048	Yes
**A4. Immediate Echolalia**	10	4.43	2.05, 9.55	0.0024	Yes
**A5. Stereotyped/Idiosyncratic Use of Words or Phrases**	15	9.06	4.38, 18.74	<0.0001	Yes

These items were 4–9 times more likely to be unscored compared to other BOSA items, suggesting they require specific attention during telehealth assessments conducted under suboptimal network conditions.

#### 3.2.2. Stream quality group comparison.

We compared the frequency of unscored items between Group A (poor stream quality: Conditions 1 + 2) and Group B (better stream quality: Conditions 3 + 4) for the vulnerable BOSA items identified in Analysis 1 (see Table 3). Items B10, B4, and A4 demonstrated significant differences in the frequency of unscored entries between the two groups, with poorer stream quality leading to more unscored items.

**Table 3:** Comparison of unscored frequencies for vulnerable BOSA items between poor (Group A) and better (Group B) stream quality. Items B10, B4, and A4 were significantly more often unscored under poor quality, while A5 showed no group difference.

Item	Group A Observed Unscored	Group B Observed Unscored	Odds Ratio (OR)	95% CI	Adjusted p-value	Significant
**B10. Spontaneous Initiation of Joint Attention**	12	2	14.40	2.32, 89.29	0.0129	Yes
**B4. Integration of Gaze and Other Behaviors During Social Overtures**	10	1	18.57	1.95, 176.49	0.0271	Yes
**A4. Immediate Echolalia**	9	1	14.62	1.55, 138.19	0.0356	Yes
**A5. Stereotyped/Idiosyncratic Use of Words or Phrases**	8	7	0.89	0.22, 3.66	1.0000	No

Notably, item A5 showed no significant difference between the groups, suggesting its vulnerability may stem from factors beyond stream quality alone.

#### 3.2.3. Quantifying the stream quality-assessment relationship/ stream quality-unscored items correlation.

Spearman’s correlation analysis confirmed a significant moderate-to-strong negative relationship (ρ = −0.58, p = 0.000646) between stream quality and unscored items. This indicates that as stream quality deteriorates, the number of unscored assessment items increases predictably, providing a quantifiable metric of how network conditions impact overall assessment completeness.

The strength and significance of this relationship highlight the systematic impact of technical network conditions on clinical assessment completeness, confirming that optimal stream quality supports more comprehensive and reliable BOSA assessments.

#### 3.2.4. Resistance of core BOSA items to stream quality variation.

Our final analysis used logistic regression to determine whether stream quality directly influences the scorability of BOSA items used in calculating the total diagnostic score. We analyzed 25 BOSA items, excluding four items showing perfect separation (B14, B15, D3, and E1), meaning their scorable status was entirely predicted by stream quality.

Individual item analyses revealed that B10, B4, and A4 showed sensitivity to stream quality before adjustment (raw p = 0.007, 0.008 and 0.016, respectively), but none remained significant after Bonferroni correction (B10: OR = 0.30, 95% CI [0.13, 0.72], adjusted p = 0.149; B4: OR = 0.25, 95% CI [0.09, 0.69], adjusted p = 0.164; A4: OR = 0.31, 95% CI [0.12, 0.81], adjusted p = 0.342). For all three, odds ratios below 1 indicated that higher stream quality was associated with lower odds of an item being coded unscorable. Odds ratios with 95% confidence intervals for all 21 analyzed items are provided in S2 Table; intervals were wide, reflecting the limited number of unscored events per item. The remaining 18 items showed no significant association between stream quality and scorability even before correction. This crucial finding suggests that the BOSA’s core diagnostic items maintain their reliability across varying network conditions, supporting the feasibility of telehealth autism assessment even in challenging technical environments.

#### 3.2.5. Inter-rater consistency under mild degradation.

To estimate inter-rater consistency under mild degradation, we compared scores assigned by independent raters to the two mildest conditions (Conditions 3 and 4) for all 10 patients, yielding 250 item-level comparisons. Total BOSA scores showed excellent agreement (ICC = 0.93), with 8 of 10 patients receiving identical total scores across conditions (mean absolute difference = 0.4 points). Item-level exact agreement was 86.4% (Cohen’s κ = 0.76), indicating substantial concordance. Agreement on item scorability, whether an item was scored or coded as unscorable, was 96.4% (κ = 0.69). Of the nine scorability discrepancies, eight involved an item coded as unscorable under Condition 3 but scored under Condition 4, consistent with a residual effect of freeze frequency on individual item scorability even at mild degradation levels. These findings are consistent with the inter-rater agreement reported for the same team of clinical psychologists in a prior BOSA validation study using the online diagnostic protocol (Cohen’s κ = 0.66, 79.8% agreement; Stroupková et al., 2024), and suggest that scorer consistency in the present study was at least comparable. However, these values should not be interpreted as a formal inter-rater reliability assessment, since they reflect agreement across two mildly different degradation conditions rather than across identical recordings. They are therefore best understood as an estimate of scoring consistency under mild degradation; a formal inter-rater reliability assessment using identical recordings remains a priority for future work.

## 4. Discussion

This study quantified how network quality shapes the reliability of BOSA in remote autism assessment. Across laboratory simulations and clinical recordings, diagnostic validity was preserved when Wi-Fi RSSI > −70 dBm and packet loss < ~0.04%. Breaching either limit produced a dose-dependent decline, which was reflected as 1-second freezes rising from ~1 every 16.7 s to ~1 every 5.0 s, and four micro-temporal items–B10 (Spontaneous Joint Attention), B4 (Integration of Gaze and Behavior), A4 (Immediate Echolalia), and A5 (Stereotyped Language)–became 4–9 times more likely to be coded “unscorable.” Yet the aggregate BOSA risk score remained stable, indicating that BOSA can still flag high-likelihood cases under moderate technical stress while signaling when in-person follow-up is needed.

By translating prior qualitative references to “connection quality” into quantitative cutoffs, we extend earlier tele-diagnostic reports that flagged connectivity as a barrier but did not specify operational thresholds [1,7]. Because modern platforms adapt bitrate, once minimal throughput is available, headline speed is a weak predictor of scorability; sub-percent packet loss produces bursty frame drops that adaptive codecs cannot fully mask as loss approaches ~0.03–0.04%, driving freeze frequency up.

BOSA items A4 and A5 (Immediate Echolalia, Stereotyped/Idiosyncratic Use of Words or Phrases) may be especially complex to interpret in telehealth contexts. According to ADOS-2/BOSA conventions, they are scored as 8 if the child produces fewer than five words spontaneously. Thus, some unscorable ratings in our dataset may reflect the child’s language level rather than technical degradation. At the same time, under suboptimal network conditions, short vocalizations can be missed by even a single freeze, leading to the same outcome. This overlap between scoring rules and technical artifacts suggests that language-related BOSA items require careful interpretation in remote settings, particularly for minimally verbal children.

Similar challenges in judging eye contact and brief vocalizations have been reported in earlier studies, although without quantifying the underlying network stress that triggers them [2,10]. Our findings clarify that the bottleneck is temporal resolution rather than social complexity: when frame cadence stutters, raters lose the micro-behavioral cues first while more global behaviors (e.g., sustained affect, turn-taking) remain visible. Practically, this suggests a triage workflow: if real-time monitoring shows freeze frequency edging into the high-risk band, clinicians should focus observation on core algorithm items that tolerate lower temporal fidelity and schedule an in-person or high-bandwidth follow-up to capture gaze-synchrony and immediate-echo phenomena in borderline cases [11,12].

Notably, the loss of micro-temporal items did not compromise the total BOSA algorithm score. Because the algorithm pools 20 + behaviors–many of which are temporally coarse–it can tolerate a handful of “8” codes without shifting the diagnostic threshold, a built-in redundancy echoed in tele-ADOS concordance studies that report 85–90% agreement with in-person verdicts under stable links [2,6,10,13,14]. This resilience underpins a pragmatic workflow: use BOSA as the first-line screen when packet-loss and Wi-Fi metrics fall inside the safe band; if real-time monitoring shows freeze frequency drifting above one per 10 s or more than two micro-items go unscored, pivot to an in-person (or high-bandwidth hub) evaluation. Such a triage model preserves the speed and reach of telehealth while protecting borderline cases from misclassification.

These findings may inform practical safeguards for telehealth-based autism assessment. A simple pre-session checklist, including verification of Wi-Fi RSSI ≥ –70 dBm and a brief packet-loss test (< 0.04%) before each BOSA appointment, could help identify technically high-risk sessions before clinical time is invested. If either threshold is not met, clinicians could consider remediation steps such as switching to a wired connection, changing location within the home, improving router proximity, or redirecting the session to a higher-bandwidth setting. During the session itself, technical monitoring could complement clinician judgment: if multiple time-sensitive items, such as B10, B4, A4, or A5, become unscorable early in the observation, the session may require technical adjustment, repetition under improved conditions, or in-person follow-up. Beyond clinician-driven monitoring, similar thresholds could be incorporated into telehealth platform design through automated pre-call diagnostics that flag suboptimal conditions and prompt remediation. Although these proposed thresholds require validation in larger and more naturalistic samples, they offer an initial framework for defining minimum technical conditions for tele-autism evaluations and may help inform future professional or institutional recommendations.

In places with poor broadband these thresholds are rarely met. Counties in the lowest broadband quintile schedule 40% fewer telehealth visits and report packet-loss rates two to three times higher than urban benchmarks [5]. Caregivers in these areas still report high satisfaction with remote options–largely due to saved travel time–but provider satisfaction drops when technical or setup difficulties occur [11]. Low-cost safeguards can narrow this gap: loaner hotspots or community telehealth hubs must be provisioned to deliver ≥ –65 dBm and < 0.03% loss; practical steps include providing larger monitors, portable speakers, and using wired internet connections or improved room setups to enhance audiovisual quality [1]; and although the telemedicine model was not initially billable, it could potentially facilitate insurance or private pay reimbursement [15]. By embedding these safeguards, bandwidth becomes a controllable aspect of care rather than a barrier determined by social circumstances.

This study has several strengths, but also limitations. Strengths include (i) a controlled packet-loss test-bed that injected identical freeze patterns across clips, (ii) precisely controlled video degradation, and (iii) item-level statistics that pinpoint which behaviors fail first. The study was designed as an exploratory pilot, intended to establish the first quantitative thresholds for a phenomenon previously described only in qualitative terms, and to generate testable parameters for confirmatory multi-site work. The modest sample of 40 degraded videos derived from 10 children across four conditions reflects the feasibility of single-centre recruitment of in-patient children undergoing ADOS-2/BOSA assessment within the study window, combined with the labor-intensive scoring procedure required from blinded clinical psychologists. This sample size limits statistical power for item-level analyses and contributes to the wide confidence intervals observed in Tables 2 and 3, and smaller effects may have gone undetected. At the same time, the consistent dose-response pattern across degradation conditions and the convergent findings from multiple statistical approaches (Fisher’s exact tests, Spearman correlation, logistic regression) support the robustness of the principal results. Relatedly, the reliability analysis reported in Section 3.2.5 compared independent ratings of mildly different degradation conditions rather than identical recordings and should therefore be interpreted as an approximation of scoring consistency rather than as formal inter-rater reliability. The specific network thresholds (–70 dBm, 0.04% packet loss) should be regarded as initial estimates, to be refined and validated in larger multi-site cohorts. Four items (B14, B15, D3, E1) exhibited perfect separation and were excluded from regression.

A second set of limitations concerns the fidelity of the simulation to real-world network instability. Laboratory freezes cannot capture every nuance of real packet loss; in particular, real Zoom sessions also produce inter-freeze artifacts such as pixelation, resolution drops, and audio desync (S1 Table) that may compound the clinical impact in ways not modeled here. Real packet loss occurs in temporally irregular bursts rather than the evenly spaced intervals used here, and live Zoom sessions degrade audio and video independently, with audio typically preserved at the expense of video, whereas our simulation freezes both streams together. These simplifications were necessary to maintain reproducibility and ensure direct comparability across degradation conditions, but they also mean that the thresholds reported here represent a controlled approximation of real-world telehealth conditions rather than a direct mirror of them. Validation against live network sessions, ideally with parallel monitoring of audio and video stream quality, remains a priority for future work. Cellular-only connections were not considered. Administration-side latency, which could affect the clinician’s delivery of instructions during live BOSA sessions, was also not modeled; however, its clinical impact is likely limited as instructions can be repeated without affecting the spontaneous behaviors that BOSA captures.

Future work should recruit multi-site cohorts stratified by network quality–including cellular-only homes–to confirm or refine the –70 dBm/ 0.04% cut-offs. Codec-level innovations (e.g., packet-loss-concealment tuned for micro-behavior detection) and automated pre-call diagnostics for lighting, audio, and camera stability should be explored [7,16]. Focused studies on the four perfectly separated items could reveal whether hardware upgrades, rater training, or algorithm tweaks best restore their scorability, particularly in children with co-occurring language or intellectual impairments.

By turning the vague concept of “adequate bandwidth” into two measurable cut-offs and a simple triage workflow, this study equips tele-autism tools to deliver fast, equitable, and evidence-based care–without sacrificing diagnostic confidence under unstable network conditions.

## 5. Conclusion

Our data show that remote autism assessments can remain clinically reliable even when bandwidth is less than ideal, provided two network minima are met: Wi-Fi power above –70 dBm and packet-loss below 0.04%. Once either threshold is breached, video freezes occur every 5–16 seconds and time-sensitive behaviors–gaze shifts, joint attention, brief vocal echoes–become increasingly unscorable. Most core diagnostic items, however, still withstand moderate degradation, allowing clear-cut cases to be identified while signaling when an on-site follow-up is needed. In practice, a one-minute tech check for signal strength and packet loss, plus real-time freeze monitoring, can turn those limits into actionable gate-keeping criteria. These thresholds should be regarded as preliminary estimates derived from a small pilot sample and require validation in larger, more diverse cohorts before broader clinical implementation. With such safeguards, and with concerted efforts to extend adequate connectivity to underserved areas, telehealth can offer a feasible, though not yet universal, pathway to timely autism diagnosis under suboptimal network conditions, even in rural areas.

## Supporting information

S1 Fig Isolated Testbed Schematic. The figure depicts the isolated testbed used for controlled network parameter testing. A source laptop streams pre-recorded clinical video via VLC through a UTP cable to a media converter, which transmits data over an optical fiber link to a second media converter. The destination laptop receives the stream via UTP. This setup allowed precise manipulation of network conditions, lagging, dropping, and duplicating packets, while maintaining patient confidentiality and avoiding public networks.
(PNG)

S1 Table Packet Dropping Impact on Video Streaming. This table summarizes the effects of progressively increasing packet-loss probabilities, introduced via the Clumsy 0.3 application, on video stream quality in our isolated optical-fiber testbed. The “Chance (%)” column indicates the probability of packet loss, while “Period” shows the approximate duration over which artifacts were observed. The “Results” column describes the severity and extent of visual artifacts, ranging from no perceptible impact (0.001%) to complete image freezing and unrecognizable video content (≥0.45%). These data informed the selection of realistic freeze patterns for subsequent BOSA scoring simulations.
(DOCX)

S2 Table Per-item logistic regression of stream quality predicting item unscorability. Binary logistic regression was fitted separately for each BOSA item, with stream quality (Code 1–4, ordinal) as the sole predictor and item scorability (unscored = 1, scored = 0) as the outcome. Odds ratios (OR) below 1 indicate that higher stream quality was associated with lower odds of an item being coded unscorable. Four items (B14, B15, D3, E1) were excluded due to perfect separation (no unscored events). P-values were adjusted using the Bonferroni correction across the 21 analyzed items. Confidence intervals are wide, reflecting the limited number of unscored events per item and the modest sample size; results should be interpreted as exploratory.
(DOCX)
