# Supplementary material for: Telehealth autism evaluation under suboptimal network conditions: Technical parameters and clinical implications
Source: PLoS One. 2026 Sep 9;21(9):e0357146. doi: 10.1371/journal.pone.0357146 (PMC13557373; doi:10.1371/journal.pone.0357146)
Supplement: Supplementary file 1 [file pone.0357146.s002.docx]

| **Chance (%)** | **Period** | **Results (impact on video stream)** |
| --- | --- | --- |
| 0.001 | — | No impact |
| 0.01 | ~25 s | Tiny artefacts, no major impact on quality |
| 0.02 | ~18 s | Irregular tiny artefacts |
| 0.025 | ~18 s | Up to 1/4 of the image has artifacts - irregularly |
| 0.03 | ~11 s | Minor artifacts, irregular artifacts in up to 3/4 of the image |
| 0.035 | ~10 s | Minor artifacts, irregular artifacts in up to 3/4 of the image |
| 0.04 | ~6 s | Minor artifacts, irregularly 1/3 of the image is unrecognizable |
| 0.05 | ~1 s | Artefacts are present almost all the time during the stream, faster movements = blurring of the images, faces cannot be recognized. |
| 0.065 | ~1 s | Artefacts at all times during transmission, in places no person or movement can be detected. |
| 0.15 | ~1 s | Cannot recognize what is happening in the video in 1/3 to 2/3 of the image, full blur, image freezing. |
| 0.45 | — | Video cannot be evaluated; artifacts associated with image freezing. |
| 0.65 | — | Video cannot be evaluated; artifacts associated with image freezing. |

**Table S1. Packet Dropping Impact on Video Streaming.**

This table summarizes the effects of progressively increasing packet-loss probabilities, introduced via the Clumsy 0.3 application, on video stream quality in our isolated optical-fiber testbed. The “Chance (%)” column indicates the probability of packet loss, while “Period” shows the approximate duration over which artifacts were observed. The “Results” column describes the severity and extent of visual artifacts, ranging from no perceptible impact (0.001%) to complete image freezing and unrecognizable video content (≥0.45%). These data informed the selection of realistic freeze patterns for subsequent BOSA scoring simulations, allowing us to model clinically relevant streaming degradation.
