# Supplementary material for: Telehealth autism evaluation under suboptimal network conditions: Technical parameters and clinical implications
Source: PLoS One. 2026 Sep 9;21(9):e0357146. doi: 10.1371/journal.pone.0357146 (PMC13557373; doi:10.1371/journal.pone.0357146)
Supplement: Supplementary file 2 [file pone.0357146.s003.docx]

| **Item** | **Unscored (n)** | **Odds Ratio** | **95% CI** | **P-value** | **Adjusted p-value** |
| --- | --- | --- | --- | --- | --- |
| B5 | 2 | 0.71 | 0.18–2.77 | 0.6208 | 1 |
| B7 | 1 | 0.72 | 0.11–4.77 | 0.7308 | 1 |
| B8 | 2 | 0.38 | 0.06–2.37 | 0.3004 | 1 |
| B9 | 4 | 1.39 | 0.54–3.60 | 0.4956 | 1 |
| B10 | 14 | 0.30 | 0.13–0.72 | 0.0071 | 0.1491 |
| B11 | 1 | 0.72 | 0.11–4.77 | 0.7308 | 1 |
| B12 | 5 | 0.54 | 0.20–1.45 | 0.2222 | 1 |
| B13a | 2 | 1.10 | 0.31–3.91 | 0.8837 | 1 |
| B14 | 0 | perfect separation | | | |
| A6 | 4 | 0.52 | 0.17–1.58 | 0.2483 | 1 |
| B1 | 2 | 1.71 | 0.44–6.70 | 0.4433 | 1 |
| B3 | 2 | 1.71 | 0.44–6.70 | 0.4433 | 1 |
| B4 | 11 | 0.25 | 0.09–0.69 | 0.0078 | 0.1638 |
| B15 | 0 | perfect separation | | | |
| C1 | 1 | 0.72 | 0.11–4.77 | 0.7308 | 1 |
| C2 | 1 | 1.67 | 0.25–11.14 | 0.5939 | 1 |
| A3 | 5 | 1.01 | 0.43–2.37 | 0.9777 | 1 |
| A4 | 10 | 0.31 | 0.12–0.81 | 0.0163 | 0.3423 |
| A5 | 15 | 1.02 | 0.55–1.91 | 0.9508 | 1 |
| D2 | 2 | 1.10 | 0.31–3.91 | 0.8837 | 1 |
| D3 | 0 | perfect separation | | | |
| D5 | 3 | 0.48 | 0.13–1.80 | 0.274 | 1 |
| D4 | 1 | 0.72 | 0.11–4.77 | 0.7308 | 1 |
| D1 | 2 | 0.38 | 0.06–2.37 | 0.3004 | 1 |
| E1 | 0 | perfect separation | | | |

**Table S2. Per-item logistic regression of stream quality predicting item unscorability.**

Binary logistic regression was fitted separately for each BOSA item, with stream quality (Code 1–4, ordinal) as the sole predictor and item scorability (unscored = 1, scored = 0) as the outcome. Odds ratios (OR) below 1 indicate that higher stream quality was associated with lower odds of an item being coded unscorable. Four items (B14, B15, D3, E1) were excluded due to perfect separation (no unscored events). P-values were adjusted using the Bonferroni correction across the 21 analyzed items. Confidence intervals are wide, reflecting the limited number of unscored events per item and the modest sample size; results should be interpreted as exploratory.
